# PKC**α** and ER**β** Are Associated with Triple-Negative Breast Cancers in African American and Caucasian Patients

**DOI:** 10.1155/2012/740353

**Published:** 2012-02-26

**Authors:** Debra A. Tonetti, Weihua Gao, Diana Escarzaga, Kelly Walters, April Szafran, John S. Coon

**Affiliations:** ^1^Department of Biopharmaceutical Sciences, College of Pharmacy, University of Illinois at Chicago, 833 S. Wood Street, Chicago, IL 60612, USA; ^2^Design and Analysis Core, Center for Clinical and Translational Science, University of Illinois, 914 S. Wood Street, Chicago, IL 60612, USA; ^3^Department of Pathology, Rush University Medical Center, 1643 W. Congress Parkway, Murdock Building, Street 593, Chicago, IL 60612, USA

## Abstract

Although the incidence of breast cancer in the United States is higher in Caucasian women compared with African American women, African-American patients have more aggressive disease as characterized by a higher percentage of triple-negative breast cancers (TNBCs), high-grade tumors, and a higher mortality rate. PKC**α** is a biomarker associated with endocrine resistance and poor prognosis and ER**β** is emerging as a protective biomarker. Immunohistochemical analysis of ER**β** and PKC**α** expression was performed on 198 formalin-fixed paraffin-embedded primary infiltrating ductal carcinomas from 105 African-American and 93 Caucasian patients. PKC**α** is positively correlated with TNBC in patients of both races and with high tumor grade in African-American patients. Patients with TNBC express less nuclear ER**β** compared with all other subtypes. We find no difference in frequency or intensity of PKC**α** or ER**β** expression between African-American and Caucasian patients. PKC**α** and ER**β** are discussed as potential therapeutic targets for the treatment of patients with TNBC.

## 1. Introduction

Although African American women have a lower incidence of breast cancer than Caucasians, repeated studies have shown that they suffer from more aggressive disease characterized by diagnosis at an earlier age, later stage, higher grade, and greater mortality [1–4]. While socioeconomic factors contribute in part to this disparity in survival, they do not account for all differences noted between these two racial groups [3, 5, 6]. In particular, premenopausal African American women present with a higher incidence of triple-negative breast cancer (TNBC), a molecular subtype that has limited targeted therapeutic options [3, 7]. Current investigations are focused upon the identification of new therapeutic targets specific to the aggressive TNBC form of breast cancers found more frequently in young African American women and the development of more effective treatment modalities.

One potential biomarker contributing to the aggressive nature of this disease in African American women is protein kinase C*α* (PKC*α*). PKC is a serine/threonine protein kinase family of enzymes comprised of at least 12 isozymes that regulate numerous cellular functions [8]. PKC*α* in particular is involved in cell migration, apoptosis, differentiation, and proliferation and plays a critical role in several disease processes including cancer [9]. Overexpression of PKC*α* is a marker of poor prognosis of breast cancers and is associated with antiestrogen resistance, ER*α*-negative tumors, and tumor aggressiveness [10–13]. Therefore, differential expression of PKC*α* may underpin the observed racial disparity in breast cancer and may be a potential therapeutic target.

Although the clinical significance of estrogen receptor *β* (ER*β*) in breast cancer is not yet firmly established, differential expression of ER*β* in breast cancers between racial groups may provide further insight [14]. Recent reports suggest ER*β* isoform expression and subcellular localization may correlate with endocrine response and breast cancer outcome [15–18]. When coexpressed with ER*α*, ER*β* appears to dampen the proliferative program of ER*α* bound to estradiol and is generally considered to be antiproliferative [19, 20]. However, understanding the effects of ER*β* is complicated by the fact that several ER*β* isoforms exist, named ER*β* 1–5 [21], and they have different implications in breast cancer development and progression. While most studies conclude that ER*β* confers a good prognosis [16] and is predictive of response to tamoxifen [22], others report association with more aggressive disease and decreased overall survival [15, 23]. The accumulated evidence thus far indicates that although ER*β* expression may predict good prognosis, the expression in relation to breast cancer subtypes and subcellular localization may influence the effect upon prognosis.

Since African American patients have more aggressive disease and lower overall survival than Caucasian patients, we tested the hypothesis that breast cancers from African American patients have higher PKC*α* expression and lower nuclear ER*β* expression and/or higher cytoplasmic ER*β* expression. We analyzed 198 primary invasive ductal carcinomas from African American and Caucasian patients for expression of PKC*α* and ER*β* to determine whether differential expression of PKC*α* and/or localization of ER*β* differed in breast cancers from African American and Caucasian women.

## 2. Materials and Methods

### 2.1. Patient Population

PKC*α* and ER*β* expression was determined by immunohistochemical (IHC) staining of 198 formalin-fixed, paraffin-embedded primary infiltrating ductal carcinomas from 105 African American and 93 Caucasian patients from the Department of Pathology, Rush University Medical Center. Complete clinicopathological characteristics were obtained from the pathology reports and the number of evaluable patients for each characteristic is given in Table 1. This study was approved by the Institutional Review Board at Rush University Medical Center and the University of Illinois at Chicago. All specimens were obtained retrospectively and posed minimal risk; therefore informed consent was waived.

### 2.2. Immunohistochemical Staining for PKC*α* and ER*β*

IHC was performed on 5 *μ*M sections of formalin-fixed paraffin-embedded tissue with the Ventana Benchmark automated staining platform using the iView DAB detection kit according to company protocol using CC1 Standard antigen retrieval. The PKC*α* antibody (rabbit polyclonal, Santa Cruz Biotechnology, sc-208) was previously validated [11] and used at a dilution of 1 : 200 and incubated at 37°C for 30 minutes. The ER*β* mouse monoclonal antibody 14C8 (Novus Biologicals Inc., Littleton, CO) was previously validated [24] and used at a 1 : 100 dilution and incubated for 30 min with HRP-rabbit Envision. This ER*β* monoclonal antibody recognizes all isoforms of ER*β* known to be expressed in breast cancer. Frequency and intensity of PKC*α* and ER*β* staining of all tumor cells on each slide were scored on a scale of 0 to 4 without knowledge of clinical patient data. Frequency of positive staining in less than 1% of tumor cells was scored as 0, 1%–10% as 1, 11% to-35% as 2, 36%–70% as 3, and over 70% as 4. A composite score is also reported based on the Allred scoring system which is a sum of the frequency and intensity scores yielding numerical values from 0 to 8 [25].

### 2.3. Statistical Analysis

We analyzed the expression of ER*β* and PKC*α* by comparing them with prognostic factors such as age, tumor grade, subtypes, and race. Chi-square tests were used for testing association between race and prognostics factors. For univariate analysis, nonparametric tests were conducted for nonnormal data. Wilcoxon Rank Sum test was performed for two groups' comparisons and Kruskal-Wallis test was performed for more than two groups' comparisons. Median, minimum, and maximum along with *P* values were reported. For multivariate analysis, to take into account prognostic factor effects, general linear regression was conducted. The interaction effects of race by prognostic factors were examined. *P* values were reported based on the type III sum of squares. *P* value < 0.05 was considered to be statistically significant. Freq, UNIVARIATE, NPAR1WAY, and GLM procedures in SAS version 9.2 (Cary, NC) were used in these analyses.

## 3. Results

### 3.1. PKC*α* and ER*β* Expression in Tumors from African American and Caucasian Breast Cancer Patients

**Figure 1:**
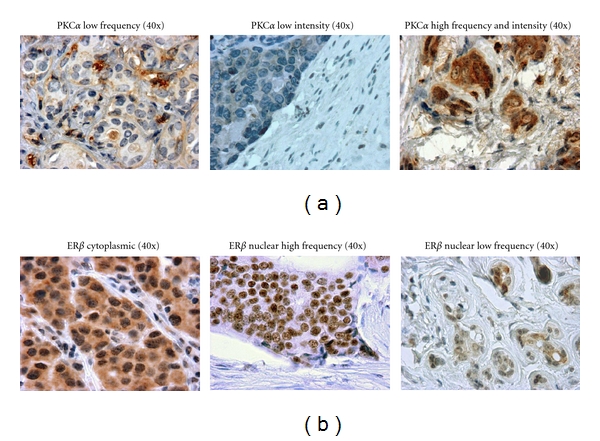
(a) Expression of PKC*α* (brown immunoperoxidase stain, blue hematoxylin counterstain). (b) Expression of ER*β* (brown immunoperoxidase stain, blue hematoxylin counterstain).

 Since PKC*α* overexpression and ER*β* expression and localization are reported to be associated with more aggressive breast cancers, we first asked whether these markers are differentially expressed based on race. Upon examination of breast cancers from 93 Caucasian and 105 African American patients, we evaluated both frequency and intensity of PKC*α* and ER*β* immunostaining in addition to subcellular localization of ER*β*. Cases exhibiting both high and low frequency and intensity of PKC*α* and ER*β* were evident including both nuclear and cytoplasmic ER*β* localization (Figures 1(a) and 1(b)). Examination of the total patient population (Table 1) revealed that 78% of all patients were positive for PKC*α* cytoplasmic staining. When patients were stratified by race, 76% of tumors from Caucasian patients and 79% of African American patients stained positively for PKC*α*. There was no statistical difference in the incidence of PKC*α* expression between races. Sixty-nine percent of patients stained positive for ER*β* including nuclear and/or cytoplasmic staining. Of these ER*β* positive cases, 57% exhibited only nuclear ER*β* staining, 20% only cytoplasmic staining, and 23% both nuclear and cytoplasmic staining. When stratified by race, there is no statistical difference in the incidence of ER*β* expression. As anticipated, there is a statistically significant difference in ER*α* expression between races reflecting the higher proportion of ER*α*-negative tumors in the African American patient population. We also observed larger tumors and more lymph node positive cases in the African American population. When the intensity and frequency of PKC*α* and ER*β* was compared by race, there was no difference in IHC staining between breast cancers from African American and Caucasian patients (Tables 2(a) and 2(b)).

### 3.2. PKC*α* Expression in ER*α*-Negative and Triple-Negative Breast Cancers

 We and others previously reported the inverse relationship between PKC*α* and ER*α* expression [12, 26, 27]. Upon stratification by race (Table 3(a)), the intensity of PKC*α* expression achieves statistical significance in the African American patients, whereas the frequency of expression does not. Conversely in the Caucasian patients, PKC*α* frequency of expression achieves statistical significance, whereas intensity of staining does not. The composite score as determined by the sum of frequency and intensity achieves statistical significance only in the African American population. When Caucasian and African American patient populations are combined, there is a statistically significant inverse relationship between PKC*α* and ER*α* frequency and intensity of expression (Table 3(b)).

We next examined PKC*α* expression stratified by breast cancer subtype categorized as luminal A (ER*α*+/PR+/Her2−), luminal B (ER*α*+/PR+/Her2+), HER2 (ER*α*−/PR−/Her2+), and triple-negative breast cancer (TNBC, ER*α*−/PR−/Her2−) based solely on receptor expression as determined by IHC. There is a strong association of PKC*α* expression and breast cancer subtypes (*P* < 0.001) that is maintained when stratified by race (see supplemental Tables 1 (a) and 1 (b) in Supplementary Material available online at doi:10.155/2012/740353). Since the incidence of TNBC is higher in African American patients compared to Caucasian patients, we asked whether PKC*α* expression is associated with TNBCs. Combining all patients we find a strong association of PKC*α* expression with the TNBC subtype compared to all other subtypes (Table 4(a)). When stratified by race, frequency of PKC*α* expression but not intensity is similarly associated with the TNBC subtype in African American and Caucasian patients (Table 4(b)).

 To determine whether TNBC is an independent predictor of PKC*α*, we performed general regression analysis with adjustment for tumor grade, patient age, lymph node status, and tumor size and found that the frequency and intensity of PKC*α* expression no longer correlates with TNBC (freq, *P* = 0.262; int, *P* = 0.957). This prompted us to ask whether PKC*α* expression correlates with the other known independent predictors of TNBC (tumor grade, patient age, lymph node status, and tumor size). Combining all patients, we find that grade 3 tumors have the highest frequency and intensity of PKC*α* expression (Table 5(a)); however there is no correlation of PKC*α* with patient age, tumor size, or lymph node status. Interestingly, when the patients are stratified by race, the positive relationship of PKC*α* and tumor grade is statistically significant only in the African American patients, but not in tumors from Caucasian patients (Table 5(b)).

 In our population of TNBC cases, we found a statistically significant correlation with tumor grade (*P* < 0.0001), patient age (*P* = 0.002), and tumor size (*P* = 0.004); however there is no correlation between TNBC and lymph node status (*P* = 0.1334).

### 3.3. ER*β* Expression and Localization in Triple-Negative Tumors

 Since both expression and subcellular localization of ER*β* are reported to influence clinical outcome and response to therapy, we examined whether ER*β* is differentially expressed in the various breast cancer subtypes. Upon stratification of all patients by subtype as previously categorized, we find there is no association of ER*β* with any particular subtype (supplemental Table 2). However when we compared TNBC to all other subtypes, we find that nuclear ER*β* expression is lower in TNBC compared to all other subtypes (Table 6). Interestingly when patients are stratified by age (<50 yrs versus ≥50 yrs), the inverse relationship of nuclear ER*β* with TNBC is statistically significant only in the younger patients (freq, *P* = 0.021), whereas when stratified by race, statistical significance is achieved only in Caucasian patients (freq, *P* = 0.023; int, *P* = 0.015) (results not shown). No association between ER*β* and tumor grade was found. 

## 4. Discussion

 This is the first report to our knowledge to examine PKC*α* and ER*β* protein expression using IHC comparing breast cancers from Caucasian and African American patients. We chose to examine the expression of these two biomarkers since both are known to be associated with endocrine response and African American patients have a higher incidence of endocrine-resistant breast cancer. PKC*α* expression is inversely related to ER*α* status [12, 13, 27], associated with more aggressive breast cancers [13] and endocrine resistance [11, 12]. Although there is less clarity regarding the clinical relevance of ER*β*, with the availability of more reliable ER*β* antibodies, the current consensus is that ER*β* expression is associated with better prognosis [28], whereas cytoplasmic localization of the ER*β*2 isoform may indicate worse prognosis [17]. Earlier studies that utilized ER*β* mRNA expression in breast cancers yielded conflicting findings correlating ER*β* expression with good prognosis while others report association with poor prognosis [29, 30]. Although we find no difference in the expression level of PKC*α* and ER*β* comparing the two races, we find a highly significant association of PKC*α* with TNBCs (Table 4(a)). Multivariate analysis revealed that the association of PKC*α* expression with higher tumor grade is likely to account for the significant association of PKC*α* with TNBC since PKC*α* does not correlate with patient age, tumor size, or lymph node status.

The African American patients in this study exhibit a higher incidence of TNBC (28% versus 18%) and more grade 3 tumors (63% versus 41%) (Table 1). This is an intriguing finding that presents a potential therapeutic opportunity since there are few treatment options available for this aggressive breast cancer subtype. PKC*α* was targeted in breast cancer patients using the antisense compound Affinitak [31]; however since the patients were not preselected for high tumor PKC*α* expression, the response to treatment was modest. We speculate that preselection of patients with TNBC with high-grade tumors in addition to elevated PKC*α* expression may improve the response rate to a PKC*α*-directed therapy. Another potential therapeutic approach may be to revisit the administration of estradiol treatment [32, 33]. Prior to the introduction of tamoxifen, high dose estrogen and diethylstilbestrol (DES) was used to treat breast cancers with similar response rates as tamoxifen, but with greater side effects [32, 34]. A recent phase 2 randomized trial was conducted comparing 2 doses of estrogen (6 mg and 30 mg) in patients with metastatic disease resistant to aromatase inhibitor therapy [35]. The majority of these patients were ER*α* positive and the clinical benefit rate of 28-29% was similar between the two dosing regimens, whereas the number of adverse events was much lower with the 6 mg estrogen dose. With the completion of this phase 2 study, we propose that the 6 mg estrogen dose be tested in patients with PKC*α*-overexpressing TNBCs. In our T47D/PKC*α* xenograft preclinical model, we reported complete tumor regression following 17*β*-estradiol (E2) administration [36] and subsequently determined that ER*α* is likely to be required for E2-triggered tumor regression. Interestingly, our preliminary studies suggest that it is extranuclear and not nuclear ER*α* that may be most important for mediating the inhibitory signal [37]. TNBCs by definition do not express nuclear ER*α*; however pathologists do not routinely score extranuclear ER*α* since optimal clinical IHC methods for detection of extranuclear or membrane ER*α* have not yet been developed. It is possible that a subset of TNBCs may in fact express extranuclear ER*α*. With the recent focus on the clinical significance of membrane and extranuclear ER*α*, detection methods for clinical use are likely to soon become available [38]. We propose further investigation is warranted to determine whether the PKC*α*/extranuclear ER*α* pathway is a feasible therapeutic target in TNBCs.

The first study to address the role of ER*β* expression and racial disparity reported a greater decrease in the protective ER*β* in breast cancers in African American patients compared with their matched adjacent normal tissue than levels found in Caucasian patients [39]. In a follow-up study using isoform-specific ER*β* primer-probe pairs, these investigators reported higher ER*β* isoform expression in ER*α*-negative breast cancers in African American patients than in Caucasian patients [40]. This finding is in agreement with our results that African American patients have a higher percentage of ER*α*-negative/ER*β*-positive breast cancers (Table 1, Caucasian, 17% ER*α*−/ER*β*+, African American, 30% ER*α*−/ER*β*+). Interestingly patients with ER*α*-negative/ER*β*-positive breast cancers are associated with increased survival compared to patients with ER*α*-negative/ER*β*-negative breast cancers [41], suggesting that these ER*α*-negative patients would benefit from tamoxifen treatment. Although we hypothesized that African American patients would have higher cytoplasmic ER*β* expression, in fact we find no difference in the level of cytoplasmic ER*β* comparing the two races (Table 2(b)). However 29% of African American patients exhibit both nuclear and cytoplasmic ER*β* expression whereas only 11% of Caucasian patients express ER*β* in both subcellular locations (Table 1). The finding that nuclear ER*β* is not associated with TNBC supports the observation that nuclear localization of ER*β* is associated with better prognosis (Table 6(b)). However, since the 14C8 antibody recognizes all isoforms of ER*β*, it is not possible to determine the specific presence and localization of ER*β*2, the isoform reported to be associated with worse prognosis when localized to the cytoplasm [17]. Therefore, the significance of the subcellular distribution of ER*β* with respect to prognosis cannot be determined in our study.

 For the first time this study examined the association of two potential prognostic biomarkers, PKC*α* and ER*β*, comparing African American and Caucasian patient populations. A significant limitation of our study is that we did not have access to treatment or follow-up information on these patients; therefore it was not possible to determine whether these biomarkers are associated with response to therapy, time to progression, or overall survival. Further investigation is warranted to determine the utility of PKC*α* as a potential therapeutic target and ER*β* as a potential biomarker for tamoxifen therapy in ER*α*-negative and TNBCs in patients of all races.

## 5. Conclusions

 Our findings suggest that PKC*α* is a potential therapeutic target for the treatment of ER*α*-negative disease, TNBCs, and high-grade tumors. Whereas lack of nuclear ER*β* in TNBCs may be a biomarker of poor prognosis, further investigation is warranted to determine the significance of ER*β* subcellular localization. While TNBCs occur more frequently in African American patients, all patients that present with this breast cancer subtype may benefit from the clinical application of these biomarkers. Further investigation into these potential therapeutic and prognostic approaches is warranted.

## Supplementary Material

Supplemental Table 1A shows there is a significant differential expression of PKC*α* among breast cancer subtypes luminal A, luminal B, TNBC and HER2 when patients of both races are combined.
Supplemental Table 1B indicates that the association between PKC*α* and subtypes is maintained when the patients are stratified by race.
Supplemental Table 2 indicates that there is no statistical differential ER*β* expression among breast cancer subtypes luminal A, luminal B, TNBC and HER2 when patients of both races are combined.

## Figures and Tables

**Table 1:** Clinicopathological characteristics of 198 infiltrating ductal carcinomas.

	Caucasian	African American	Total	*P* value*
	*N* (%)	*N *(%)	*N* (%)	
PKC*α*+	71 (76)	83 (79)	154 (78)	0.648
PKC*α*−	22 (24)	22 (21)	44 (22)	

ER*α*+	63 (68)	55 (52)	118 (60)	0.028**
ER*α*−	30 (32)	50 (48)	80 (40)	

ER*β*+	65 (70)	72 (69)	137 (69)	0.847
ER*β*−	28 (30)	33 (31)	61 (31)	

ER*β*+ (nuc + cyto)	11 (17)	21 (29)	32 (23)	
ER*β*+ (nuc)	41 (63)	37 (51)	78 (57)	0.221
ER*β*+ (cyto)	13 (20)	14 (19)	27 (20)	

ER*α*+/ER*β*+	49 (53)	40 (38)	89 (45)	0.108
ER*α*+/ER*β*−	14 (15)	15 (14)	29 (15)	
ER*α*−/ER*β*+	16 (17)	32 (30)	48 (24)	
ER*α*−/ER*β*−	14 (15)	18 (17)	32 (16)	

Subtype^#^				
Luminal A	32 (35)	26 (26)	58 (30)	
Luminal B	30 (33)	29 (28)	59 (31)	0.205
Her2+	13 (14)	18 (18)	31 (16)	
TNBC	16 (18)	29 (28)	45 (23)	

Grade^†^				
1	6 (13)	6 (8)	12 (10)	
2	21 (46)	21 (29)	42 (35)	0.068
3	19 (41)	46 (63)	65 (55)	

Lymph node+	28 (35)	52 (58)	80 (47)	0.0024**
Lymph node−	53 (65)	38 (42)	91 (53)	

Tumor size	2.17 (1.47)	2.97 (1.87)	2.60 (1.73)	0.0007**
Mean (SD)				

Age				
<50	28 (30)	45 (43)	73 (37)	0.064
≥50	65 (70)	60 (57)	125 (63)	

*All *P* values were calculated using the Chi-square test. **P < 0.05; ^#^Five patients categorized as ER−/PR+/Her2− were not assigned to a subtype category (3 African American, 2 Caucasian patients).

^†^Tumor grade was available on 46/93 Caucasian patients and 73/105 African American patients.

**Table:** (a)

Outcome	Median (minimum, maximum)		*P* value*
	AA	Caucasian	
PKC*α* (freq)	2 (0,4)	2 (0,4)	0.46
PKC*α* (int)	2 (0,4)	1 (0,4)	0.52
PKC*α* (sum)	4 (0,8)	4 (0,8)	0.49

*P value based on the Wilcoxon rank-(sum) test.

**Table:** (b)

Outcome	Median (minimum, maximum)		*P* value*
	AA	Caucasian	
ER*β* freq(n)	2 (0, 4)	2 (0, 4)	1.00
ER*β* int (n)	1 (0, 4)	1 (0, 4)	0.76
ER*β* freq (c)	3 (1, 4)	3 (2, 4)	0.83
ER*β* int (c)	1 (1, 3)	1 (1, 4)	0.84

*P value based on Wilcoxon rank-Sum test. n: nuclear; c: cytoplasmic.

**Table:** (a)

Race	Outcome	Median (minimum, maximum)		*P *value*
		ER(−) (*N* = 80)	ER(+) (*N* = 118)	
	PKC (freq)	2 (0, 4)	2 (0, 4)	0.09
AA	PKC (int)	2 (0, 4)	1 (0, 4)	0.01^††^
	PKC (sum)	5 (0, 8)	4 (0, 8)	0.02^†^

	PKC (freq)	3 (0, 4)	2 (0, 4)	0.02^†^
Caucasian	PKC (int)	1.5 (0, 4)	1 (0, 4)	0.38
	PKC (sum)	4.5 (0, 8)	3 (0, 7)	0.07

*P value based on the Wilcoxon Rank (sum) Test. ^†^ *P* value <0.05; ^††^ *P* value <0.01.

**Table:** (b)

Outcome	Median (minimum, maximum)		*P* value*
	ER(−)	ER(+)	
PKC*α* (freq)	3 (0, 4)	2 (0, 4)	0.004^††^
PKC*α* (int)	2 (0, 4)	1 (0, 4)	0.012^†^
PKC*α* (sum)	5 (0, 8)	3 (0, 8)	0.002^††^

*P value based on the Wilcoxon Rank (sum) Test. ^†^ *P* value <0.05; ^††^ *P* value <0.01.

**Table:** (a)

Outcome	Median (minimum, maximum)		*P* value*
	TNBC (*N* = 45)	All other subtypes (*N* = 153)	
PKC*α* (freq)	3 (0, 4)	2 (0, 4)	0.001^†††^
PKC*α* (int)	2 (0, 4)	1 (0, 4)	0.010^†^
PKC*α* (sum)	5 (0, 8)	4 (0, 8)	0.001^†††^

*P value based on the Wilcoxon Rank-(sum) test. ^†^ *P* value <0.05; ^††^ *P* value <0.01; ^†††^ *P* value <0.001.

**Table:** (b)

Race	Outcome	Median (Minimum, maximum)		*P* value*
		TNBC (*N* = 45)	All other subtypes (*N* = 153)	
AA (*N* = 105)	PKC (freq)	3 (0, 4)	2 (0, 4)	0.019^†^
	PKC (int)	2 (0, 4)	1 (0, 4)	0.061
	PKC (sum)	5 (0, 8)	4 (0, 8)	0.014^†^

Caucasian (*N* = 93)	PKC (freq)	3 (0, 4)	2 (0, 4)	0.010^††^
	PKC (int)	2 (0, 3)	1 (0, 4)	0.087
	PKC (sum)	5 (0, 7)	3 (0, 8)	0.018^†^

*P value based on the Wilcoxon Rank-(sum) test. ^†^ *P* value <0.05; ^††^ *P* value <0.01.

**Table:** (a)

Outcome	Median (Minimum, maximum)			*P* value*
	Grade = 1 (*N* = 12)	Grade = 2 (*N* = 42)	Grade = 3 (*N* = 65)	
PKC*α* (freq)	0.50 (0, 3)	2.00 (0, 4)	3.00 (0, 4)	0.010^††^
PKC*α* (int)	0.50 (0, 3)	1.00 (0, 3)	2.00 (0, 4)	0.012^†^
PKC*α* (sum)	1.00 (0, 6)	3.50 (0, 7)	5.00 (0, 8)	0.004^††^

*P value is based on the Kruskal-Wallis Test. ^†^ *P* value <0.05; ^††^ *P* value <0.01.

**Table:** (b)

Race	Outcome	Median (Minimum, maximum)			*P* value*
		Grade=1 (*N* = 6)		Grade=3 (*N* = 46)	
	PKC*α* (freq)	0 (0, 1)	2 (0, 3)	2 (0, 4)	0.007^††^
AA (*N* = 73)	PKC*α* (int))	0 (0, 3)	1 (0, 3)	2 (0, 3)	0.017^†^
	PKC*α* (sum)	0 (0, 4)	3 (0, 5)	5 (0, 7)	0.003^††^

		Grade = 1 (*N* = 6)	Grade = 2 (*N* = 21)	Grade = 3 (*N* = 19)	

	PKC*α* (freq)	2.5 (0, 3)	2 (0, 4)	3 (0, 4)	0.248
Caucasian (*N* = 46)	PKC*α* (int)	1.5 (0, 3)	2 (0, 3)	2 (0, 4)	0.277
	PKC*α* (sum)	4.0 (0, 6)	4 (0, 7)	5 (0, 8)	0.169

*P value is based on the Kruskal-Wallis Test. ^†^ *P* value <0.05; ^††^ *P* value < 0.01.

**Table 6:** Nuclear ER*β* expression is lower in triple-negative patients.

Outcome	Median (Minimum, maximum)		*P* value*
	TNBC (*N* = 45)	All other subtypes (*N* = 153)	
ER*β* (freq) (n)	0 (0, 4)	2 (0, 4)	0.022^†^
ER*β* (int) (n)	0 (0, 4)	1 (0, 4)	0.024^†^
ER*β* (freq) (c)	3 (1, 3)	3 (2, 4)	0.079
ER*β* (int) (c)	1 (1, 3)	1 (1, 4)	0.378

*P value is based on the Wilcoxon Rank-(sum) test. n: nuclear; c: cytoplasmic;

^†^ *P* value < 0.05.

## Abbreviations

E2: 17*β*-estradiol
ER: Estrogen receptor
IHC: Immunohistochemistry
PKC: Protein kinase C
TNBC: Triple-negative breast cancer.

## References

[1] Brower V (2008). Cancer disparities: disentangling the effects of race and genetics. *Journal of the National Cancer Institute*.

[2] Amend K, Hicks D, Ambrosone CB (2006). Breast cancer in African-American women: differences in tumor biology from European-American women. *Cancer Research*.

[3] Carey LA, Perou CM, Livasy CA (2006). Race, breast cancer subtypes, and survival in the Carolina breast cancer study. *Journal of the American Medical Association*.

[4] Dignam JJ (2006). The ongoing search for the sources of the breast cancer survival disparity. *Journal of Clinical Oncology*.

[5] Cunningham JE, Butler WM (2004). Racial disparities in female breast cancer in South Carolina: clinical evidence for a biological basis. *Breast Cancer Research and Treatment*.

[6] Chlebowski RT, Chen Z, Anderson GL (2005). Ethnicity and breast cancer: factors influencing differences in incidence and outcome. *Journal of the National Cancer Institute*.

[7] Stead LA, Lash TL, Sobieraj JE (2009). Triple-negative breast cancers are increased in black women regardless of age or body mass index. *Breast Cancer Research*.

[8] Rosse C, Linch M, Kermorgant S, Cameron AJ, Boeckeler K, Parker PJ (2010). PKC and the control of localized signal dynamics. *Nature Reviews Molecular Cell Biology*.

[9] Konopatskaya O, Poole AW (2009). Protein kinase Calpha: disease regulator and therapeutic target. *Trends in Pharmacological Sciences*.

[10] Frankel LB, Lykkesfeldt AE, Hansen JB, Stenvang J (2007). Protein Kinase C *α* is a marker for antiestrogen resistance and is involved in the growth of tamoxifen resistant human breast cancer cells. *Breast Cancer Research and Treatment*.

[11] Tonetti DA, Morrow M, Kidwai N, Gupta A, Badve S (2003). Elevated protein kinase C alpha expression may be predictive of tamoxifen treatment failure. *British Journal of Cancer*.

[12] Assender JW, Gee JM, Lewis I, Ellis IO, Robertson JF, Nicholson RI (2007). Protein kinase C isoform expression as a predictor of disease outcome on endocrine therapy in breast cancer. *Journal of Clinical Pathology*.

[13] Lonne GK, Cornmark L, Zahirovic IO, Landberg G, Jirstrom K, Larsson C (2010). PKCalpha expression is a marker for breast cancer aggressiveness. *Molecular Cancer*.

[14] Fox EM, Davis RJ, Shupnik MA (2008). ER*β* in breast cancer—onlooker, passive player, or active protector?. *Steroids*.

[15] Novelli F, Milella M, Melucci E (2008). A divergent role for estrogen receptor-beta in node-positive and node-negative breast cancer classified according to molecular subtypes: an observational prospective study. *Breast Cancer Research*.

[16] Honma N, Horii R, Iwase T (2008). Clinical importance of estrogen receptor-*β* evaluation in breast cancer patients treated with adjuvant tamoxifen therapy. *Journal of Clinical Oncology*.

[17] Shaaban AM, Green AR, Karthik S (2008). Nuclear and cytoplasmic expression of ERbeta1, ERbeta2, and ERbeta5 identifies distinct prognostic outcome for breast cancer patients. *Clinical Cancer Research*.

[18] Skliris GP, Leygue E, Curtis-Snell L, Watson PH, Murphy LC (2006). Expression of oestrogen receptor-*β* in oestrogen receptor-*α* negative human breast tumours. *British Journal of Cancer*.

[19] Sotoca AM, van den Berg H, Vervoort J (2008). Influence of cellular ERalpha/ERbeta ratio on the ERalpha-agonist induced proliferation of human T47D breast cancer cells. *The Journal of Toxicological Sciences*.

[20] Speirs V, Walker RA (2007). New perspectives into the biological and clinical relevance of oestrogen receptors in the human breast. *Journal of Pathology*.

[21] Moore JT, McKee DD, Slentz-Kesler K (1998). Cloning and characterization of human estrogen receptor *β* isoforms. *Biochemical and Biophysical Research Communications*.

[22] Mann S, Laucirica R, Carlson N (2001). Estrogen receptor beta expression in invasive breast cancer. *Human Pathology*.

[23] Umekita Y, Souda M, Ohi Y (2006). Expression of wild-type estrogen receptor *β* protein in human breast cancer: specific correlation with HER2/neu overexpression. *Pathology International*.

[24] Tonetti DA, Rubenstein R, DeLeon M (2003). Stable transfection of an estrogen receptor beta cDNA isoform into MDA-MB-231 breast cancer cells. *Journal of Steroid Biochemistry and Molecular Biology*.

[25] Allred DC, Clark GM, Elledge R (1993). Association of p53 protein expression with tumor cell proliferation rate and clinical outcome in node-negative breast cancer. *Journal of the National Cancer Institute*.

[26] Borner C, Wyss R, Regazzi R, Eppenberger U, Fabbro D (1987). Immunological quantitation of phopholipid/Ca2+-dependent protein kinase of human mammary carcinoma cells: inverse relationship to estrogen receptors. *International Journal of Cancer*.

[27] Fournier DB, Chisamore M, Lurain JR, Rademaker AW, Jordan VC, Tonetti DA (2001). Protein kinase C alpha expression is inversely related to ER status in endometrial carcinoma: possible role in AP-1-mediated proliferation of ER-negative endometrial cancer. *Gynecologic Oncology*.

[28] Hartman J, Strom A, Gustafsson JA (2009). Estrogen receptor beta in breast cancer-diagnostic and therapeutic implications. *Steroids*.

[29] Speirs V, Malone C, Walton DS, Kerin MJ, Atkin SL (1999). Increased expression of estrogen receptor *β* mRNA in tamoxifen-resistant breast cancer patients. *Cancer Research*.

[30] Dotzlaw H, Leygue E, Watson PH, Murphy LC (1999). Estrogen receptor-*β* messenger RNA expression in human breast tumor biopsies: relationship to steroid receptor status and regulation by progestins. *Cancer Research*.

[31] Roychowdhury D, Lahn M (2003). Antisense therapy directed to protein kinase C-alpha (Affinitak, LY900003/ISIS 3521): potential role in breast cancer. *Semin Oncol*.

[32] Ingle JN (2002). Estrogen as therapy for breast cancer. *Breast Cancer Research*.

[33] Mahtani RL, Stein A, Vogel CL (2009). High-dose estrogen as salvage hormonal therapy for highly refractory metastatic breast cancer: a retrospective chart review. *Clinical Therapeutics*.

[34] Peethambaram PP, Ingle JN, Suman VJ, Hartmann LC, Loprinzi CL (1999). Randomized trial of diethylstilbestrol vs. tamoxifen in postmenopausal women with metastatic breast cancer. An updated analysis. *Breast Cancer Research and Treatment*.

[35] Ellis MJ, Gao F, Dehdashti F (2009). Lower-dose vs high-dose oral estradiol therapy of hormone receptor-positive, aromatase inhibitor-resistant advanced breast cancer: a phase 2 randomized study. *Journal of the American Medical Association*.

[36] Chisamore MJ, Ahmed Y, Bentrem DJ, Jordan VC, Tonetti DA (2001). Novel antitumor effect of estradiol in athymic mice injected with a T47D breast cancer cell line overexpressing protein kinase C*α*. *Clinical Cancer Research*.

[37] Zhang Y, Zhao H, Asztalos S, Chisamore M, Sitabkhan Y, Tonetti DA (2009). Estradiol-induced regression in T47D:A18/PKC*α* tumors requires the estrogen receptor and interaction with the extracellular matrix. *Molecular Cancer Research*.

[38] Levin ER, Pietras RJ (2008). Estrogen receptors outside the nucleus in breast cancer. *Breast Cancer Research and Treatment*.

[39] Poola I, Clarke R, DeWitty R, Leffall LD (2002). Functionally active estrogen receptor isoform profiles in the breast tumors of African American women are different from the profiles in breast tumors of Caucasian women. *Cancer*.

[40] Poola I, Fuqua SA, De Witty RL, Abraham J, Marshallack JJ, Liu A (2005). Estrogen receptor alpha-negative breast cancer tissues express significant levels of estrogen-independent transcription factors, ERbeta1 and ERbeta5: potential molecular targets for chemoprevention.. *Clinical Cancer Research*.

[41] Gruvberger-Saal SK, Bendahl PO, Saal LH (2007). Estrogen receptor *β* expression is associated with tamoxifen response in ER*α*-negative breast carcinoma. *Clinical Cancer Research*.

