# Supplementary material for: PKCα and ERβ Are Associated with Triple-Negative Breast Cancers in African American and Caucasian Patients
Source: Int J Breast Cancer. 2012 Feb 26;2012:740353. doi: 10.1155/2012/740353 (PMC3299310; doi:10.1155/2012/740353)
Supplement: Supplementary file 1 — Supplemental Table 1A shows there is a significant differential expression of PKCα among breast cancer subtypes luminal A, luminal B, TNBC and HER2 when patients of both races are combined. Supplemental Table 1B indicates that the association between PKCα and subtypes is maintained when the patients are stratified by race. Supplemental Table 2 indicates that there is no statistical differential ERβ expression among breast cancer subtypes luminal A, luminal B, TNBC and HER2 when patients of both races are combined. [file 740353.f1.pdf]

Supplemental Table 1A. Association between PKCα and subtypes

Outcome	Median (Minimum, Maximum)				*P*-value*
	Luminal A (N=64)	Luminal B (N=66)	TNBC (N=44)	HER2 (N=24)	
PKCα (freq)	2.00 (0, 4)	2.00 (0, 4)	3.00 (0, 4)	2.50 (0, 4)	0.0004†††
PKCα (int)	2.00 (0, 4)	1.00 (0, 3)	2.00 (0, 4)	2.00 (0, 4)	0.0013††
PKCα (sum)	4.00 (0, 8)	3.00 (0, 7)	5.00 (0, 8)	4.50 (0, 8)	0.0002†††

**P*-value is based on the Kruskal-Wallis Test. †*P*-value <0.05; ††*P*-value <0.01; †††*P*-value <0.001.

Supplemental Table 1B. Association between PKCα and subtypes stratified by race

Race	Outcome	Median (Minimum, Maximum)				*P*-value*
		**A (N=64)**	**B (N=66)**	**TNBC (N=44)**	**HER2 (N=24)**	
AA	PKCα (freq)	2.0 (0, 4)	1.5 (0, 4)	3.0 (0, 4)	2.0 (0, 4)	0.0530
	PKCα (int)	2.0 (0, 4)	1.0 (0, 3)	2.0 (0, 4)	2.0 (0, 3)	0.0109†
	PKCα (sum)	4.0 (0, 8)	3.0 (0, 7)	5.0 (0, 8)	4.0 (0, 7)	0.0094††
Caucasian	PKCα (freq)	2.0 (0, 4)	2.0 (0, 4)	3.0 (0, 4)	3.0 (0, 4)	0.0093††
	PKCα (int)	1.5 (0, 4)	1.0 (0, 3)	2.0 (0, 3)	2.0 (0, 4)	0.1628
	PKCα (sum)	3.0 (0, 7)	3.0 (0, 7)	5.0 (0, 7)	5.0 (0, 8)	0.0276†

**P*-value is based on the Kruskal-Wallis Test. †*P*-value <0.05; ††*P*-value <0.01; †††*P*-value <0.001.

Supplemental Table 2. ERβ expression stratified by subtype

Outcome	Median (Minimum, Maximum)				*P*-value*
	Luminal A (N=58)	Luminal B (N=59)	TNBC (N=45)	HER2 + (N=31)	
ERβ freq (n)	2.00 (0, 4)	2.00 (0, 4)	0.00 (0, 4)	1.00 (0, 4)	0.082
ERβ int (n)	1.50 (0, 4)	2.00 (0, 4)	0.00 (0, 4)	1.00 (0, 3)	0.059
ERβ freq (c)	3.00 (2, 3)	3.00 (2, 4)	3.00 (1, 3)	3.00 (2, 3)	0.158
ERβ int (c)	1.50 (1, 3)	1.00 (1, 4)	1.00 (1, 3)	1.00 (1, 2)	0.698

**P*-value based on the Kruskal-Wallis test. n: nuclear; c: cytoplasmic; TNBC: triple negative breast cancer

#
